# HIV Disclosure—Professional Body Guidelines, the Law and the Boundaries of Medical Advice

**DOI:** 10.1093/medlaw/fwab011

**Published:** 2021-05-19

**Authors:** Samantha Ryan, Matt Phillips

**Affiliations:** 1 Newcastle Law School, 21-24 Windsor Terrace, Newcastle University, Newcastle upon Tyne, NE2 4HQ, UK; 2 North Cumbria Integrated Care NHSFT, Pillars Building, Cumberland Infirmary, Infirmary Street, Carlisle, CA2 7HY, UK

**Keywords:** Advice, disclosure, guidelines, HIV, law, professional responsibility

## Abstract

This article examines the current BHIVA/BASHH guidelines on the disclosure of HIV+ status in the context of sexual activity. It assesses whether the guidance provided on how to avoid criminal prosecution accurately reflects the prevailing position in law. Given that aspects of the guidance related to non-disclosure of HIV infection in the context of low or negligible risk are as yet untested in UK law, it is argued that there is some uncertainty as to whether the professional body guidelines and the law can be reconciled with each other. The article also considers whether the BHIVA/BASHH guidelines stray beyond the boundaries of medical advice as normally understood (focused on the protection of health and the prevention of onward transmission), by posing both as legal advice on how to avoid prosecution and offering what could be viewed as a moral judgement as to when disclosure is required. While a bio-medical assessment of risk naturally shapes clinical guidelines and may also inform views as to appropriate sexual behaviour and risk-taking, it is unclear whether scientific assessment of risk should be the sole guide when it comes to determining the nature of any disclosure obligation or the medical advice to be given on this matter.

## I. INTRODUCTION

The issue of disclosing HIV+ status to sexual partners is extremely complex. For individuals who are HIV+, the decision to disclose comes with many potential risks—the loss of privacy/confidentiality, stigma, rejection, the breakdown of relationships, loss of home or economic stability, and, for some, a very real risk of personal violence.^1^ The criminalisation of intentional and reckless transmission of HIV in England, Wales, and Scotland, combined with a lack of clarity in the law regarding precisely what is required in terms of disclosure to avoid criminal liability, has served to only further complicate this issue for those living with HIV. The potential for criminal sanctions has also added a layer of complexity to the relationship between HIV+ individuals and those involved in treating and supporting them.^2^ Ceri Evans goes so far as to say that the criminalisation of HIV transmission has ‘necessitated a change in practice and guidance from the professional bodies and, therefore, perhaps, a change in the nature of the role of healthcare professionals’.^3^

This article will focus specifically on the issue of HIV disclosure advice provided by healthcare professionals in their general capacity of advising individuals of the wider impact of illness.^4^ As Iain Brassington observes, ‘when assessing what a person ought to do or ought to have done in a given situation, we may look to the law, or to morality, or to professional codes for clarification’.^5^ It seems clear that when discussing disclosure, healthcare professionals have to operate within this triad of factors—law, morality, and professional guidelines. It is also recognised that non-disclosure raises contentious questions around responsibility, individual morality, and moral behaviour.^6^ The aim of this article is to explore whether the current professional body guidelines, which set out the advice to be given to people living with HIV, can be reconciled with the law on criminal liability for HIV transmission and exposure. It will be shown that aspects of the guidance related to non-disclosure of HIV infection in the context of low or negligible risk are as yet untested in law, and that, accordingly, there is some uncertainty as to whether the guidelines accurately reflect the prevailing legal position.

The boundaries of appropriate medical advice will also be considered. It will be suggested that the advice contained in the guidelines strays beyond the concept of medical advice as normally understood in two ways. First, by presenting itself as quasi-legal advice and, secondly, by adopting a position on non-disclosure that reflects a view, some would say a moral view, on appropriate behaviour that is not strictly speaking limited to protection of health or prevention of onward transmission. To begin, however, this article will first outline the professional responsibilities and duties owed to HIV+ patients and the wider community by medical healthcare professionals. It will then detail the current British HIV Association (BHIVA)/British Association for Sexual Health and HIV (BASHH) professional body guidelines on disclosure advice, taking account of the prevailing scientific understanding of risk in the context of HIV transmission.

## II. PROFESSIONAL RESPONSIBILITY FOR HIV+ PATIENTS, DISCLOSURE ADVICE AND PROFESSIONAL BODY GUIDELINES

The standard ethical principles and professional responsibilities that apply generally between healthcare professionals and their patients also apply in the context of the HIV+ patient (such as, the duty to treat your patient with respect, and the duty to respect a patient’s right to confidentiality, privacy, and dignity).^7^ However, it is also the case that within the specific context of HIV, some of these duties require a more nuanced consideration. By way of example, the doctor–patient relationship has always been one in which confidentiality is key, particularly in sexual health where, until recently, the Venereal Diseases Regulations 1974 prohibited sharing information. Yet, in the context of HIV, healthcare professionals’ corresponding duties to protect the wider community means that patient confidentiality may be breached in the public interest if it is believed that the patient is putting sexual contacts at risk.^8^ This also holds true for notifiable diseases, of which HIV is not one, where public health interests of knowing that someone has, for example, typhoid, outweigh the normal privileges of individual confidentiality.

In terms of specific duties owed to HIV patients and third parties, a healthcare professional has an ethical duty to ‘properly advise his or her own patient with regard to protecting others from infection’.^9^ The General Medical Council (GMC), the body which regulates doctors in the UK, states that patients should be told how they can protect themselves from infection, including the practical measures they can take to avoid transmission.^10^ The importance of informing sexual contacts about the risk of transmission of sexually transmitted serious communicable diseases is also emphasised.^11^ With regard to disclosure, the BHIVA/BASHH professional guidelines recommend that healthcare professionals commence discussions about disclosure to sexual partners after diagnosis.^12^ Disclosure should be recognised as a process (often a difficult one) and patients should be supported throughout that process.^13^ It is further recommended that issues of disclosure are revisited as circumstances change, and appropriate advice and support should be made available.^14^ Non-disclosure should be discussed sensitively and barriers to disclosure should be identified and means of overcoming them should be discussed.^15^

The issue of disclosure, particularly when mixed with discussions around potential criminal liability for transmission, can be especially challenging for healthcare professionals. The use of the criminal law to punish HIV transmission is something many healthcare professionals have expressed concern about, and having to discuss this issue with their patients leaves many feeling uncomfortable or conflicted.^16^ As noted by Evans:

Whilst it is not ‘anti-patient’ to inform them of the legal consequences of transmitting the disease they have – it is part of the information they need to receive – many healthcare professionals disagree with the use of the criminal law and feel uncomfortable with having to discuss it with their clients.^17^

Particular care needs to be taken when raising the issue of potential criminal sanction with patients. In order to maintain a positive therapeutic relationship, it is essential that reference to criminalisation is not seen as a threat.^18^ The possibility of criminal prosecution can negatively affect the relationship between patient and healthcare professional in other ways too.^19^ The fact that medical records may be used as part of the investigatory process, or in a prosecution, may cause HIV+ individuals to be less open and honest with healthcare professionals about their sexual relationships with others, for fear that this information could be used against them.^20^ The risk of criminal prosecution has also impacted on the way in which healthcare professionals approach their note taking, with some seeking to minimise note-taking in order to protect their patient, and others taking care to write down all advice given as a means of protecting themselves.^21^

Professional guidance on HIV transmission and the law, which includes consideration of disclosure requirements, can be found in guidance from the BHIVA/BASHH.^22^ As guidance, this document is not legally binding.^23^ While recognising the importance of disclosing HIV+ status to a partner before sex to support informed decision making about risk and safer sex behaviours, the guidance states that ‘To avoid successful prosecution an individual who is not taking effective antiretroviral medication (ART) and does not use a condom must disclose their HIV status to sexual partners before sex takes place’.^24^ This implies that disclosure is not required in the case of proper and consistent condom use, or adherence to effective ART, which maintains a suppressed viral load. The BHIVA/BASHH guidelines specifically state that in the event of a condom split, it is advisable to disclose HIV status so that the sexual partner can consider the need for PEPSE (post-exposure prophylaxis), and that disclosure in these circumstances would suggest that the person was not reckless.^25^

It is important to consider the scientific understanding of HIV transmission risk in the context of condom use and low viral load that lies at the heart of these clinical guidelines. The suggestion that disclosure is not necessary when condoms are used is based on research findings that when such precautions are used properly, and consistently, there is a low risk of HIV transmission.^26^ It is important to be clear as to what ‘low risk’ means in this context. In terms of risk in the context of heterosexual serodifferent couples (where one person is HIV+ and the other is not), research has shown that HIV transmission was 80% lower among couples who reported always using male condoms, as compared with couples who said they never used condoms.^27^ Other research, which also focused on condom use in the context of vaginal intercourse, found a 1 in 10,000 rate of transmission for the non-infected female sexual partner, and a 1 in 20,000 risk of transmission for the non-infected male partner.^28^ In relation to condom effectiveness at reducing HIV transmission when used by men who have sex with men (MSM), an effectiveness rate of between 70% and 80% for consistent condom use during anal sex was found,^29^ although later meta-analyses have found an even higher effectiveness rate of approximately 90%.^30^

Care must be taken, however, when interpreting these findings because it is not correct to say, for example, that for MSM there is a 20–30% chance of acquiring HIV when a condom is used. What the research shows is that the base-line risk of transmission is *reduced* by between 70% and 80% (or 90% following more recent studies) when condoms are used.^31^ So, while the per act risk of transmission for unprotected (no use of ART) receptive anal intercourse is 138 per 10,000, this figures becomes 28 per 10,000 in the context of condom use.^32^ The per act risk for unprotected receptive penile-vaginal intercourse is 8 per 10,000, which is reduced to 1.6 per 10,000 when condoms are used. It is important to note that low risk does not mean no risk, and it is clear that condom use does not completely eliminate the risk of transmitting HIV.^33^ Improper and/or inconsistent use, or condom slippage or breakage, undermines the effectiveness of condoms as a means of preventing transmission. It is for these reasons that condom use continues to be described as low risk activity.

The BHIVA/BASHH guidelines also suggest that disclosure is not necessary when the HIV+ person is taking ART and, as result, has a low viral load. A substantial amount of research into transmission risk and ART/viral load has been undertaken during the last 20 years.^34^ At the time the BHIVA/BASHH guidelines were issued, results were emerging from the PARTNER Study Phase 1. This study found no instances of HIV transmission amongst heterosexual and gay serodifferent couples who reported condomless sex when the viral load of the HIV+ partner, who was taking ART, was undetectable.^35^ However, the findings in relation to receptive anal sex, where the HIV negative partner was the receptive partner, were expressed with a note of caution: ‘appreciable levels of risk cannot be excluded, particularly for anal sex and when considered from the perspective of cumulative risk over several years’.^36^ Research findings, since the BHIVA/BASHH guidelines were issued, have overwhelmingly found (in all contexts) that there is a negligible risk of sexually transmitting HIV when a HIV+ partner adheres to ART and maintains a suppressed viral load of less than 200 copies/ml on consecutive measurements every 4–6 months.^37^

The strength of the research findings regarding a negligible risk of transmission for both heterosexual and gay serodifferent couples when the HIV+ partner has a low viral load is such that in 2016 the Prevention Access Campaign launched the U = U (undetectable equals untransmittable) campaign, which states that a person living with HIV who has an undetectable viral load does not transmit HIV to their partners.^38^ This statement has been endorsed by more than 780 HIV organisations, including the PARTNER2 study and by organisations such as BHIVA and the Terrence Higgins Trust in the UK.^39^ Chloe Orkin, the Past Chair of BHIVA, supported the U = U message and said that clear and unambiguous terminology regarding risk must be used, and recommended that terms such as ‘no risk’ or ‘zero risk’, as opposed to ‘minimal’ or ‘negligible’ risk, were employed.^40^ It is, however, important to recognise that not all HIV+ people have the same access to testing, effective treatment, viral load monitoring, and support to enable them to reach and maintain viral suppression. Thus, for some people, U = U does not reflect their lived reality.

It is suggested that by providing that disclosure is not required when the risk of transmission is low or minimal, the BHIVA/BASHH guidelines go further than a simple scientific assessment of risk or straightforward advice about preventing transmission and safer sex. It is also worth noting that the professional body guidelines are framed as quasi-legal advice, in that the recommendations are explicitly framed as a means of avoiding prosecution. Whether this advice remains within the confines of appropriate medical advice will be considered in more detail in Section IV. The prevailing legal position will now be discussed in order to determine if the BHIVA/BASHH guidelines do, in fact, reflect the law.

## III THE LAW: HIV TRANSMISSION, DISCLOSURE AND CRIMINAL LIABILITY

The basic position in England and Wales is that a person will be guilty of a criminal offence if, knowing of their HIV+ status, they either intentionally or recklessly transmit HIV to a sexual partner where that sexual partner has not given an informed consent to run the risk of infection.^41^ Actual transmission must have occurred in order for a charge of intentional or reckless transmission to be pursued, although it is possible to charge attempted transmission in certain circumstances.^42^ In Scotland, if there is evidence that the accused intentionally infected the victim, the charge would be assault.^43^ If there is no evidence of intent to cause harm but evidence that indicates criminal recklessness, then the appropriate charge is culpable and reckless conduct if transmission occurs.^44^ Culpable and reckless conduct may also be charged if someone has recklessly exposed another to the risk of infection even if transmission has not in fact occurred.^45^ Although the initial cases concerned reckless HIV transmission,^46^ subsequent cases have included intentional and reckless transmission of HIV, transmission of herpes, gonorrhoea, hepatitis B, and hepatitis C, and, in Scotland, conviction for exposure to a risk of HIV infection.^47^

In the following sections, the issue of disclosure and the way in which this has been addressed by the courts will be outlined. The legal position on recklessness, focusing mainly on the context of condom use and low viral load, will then be considered. The Crown Prosecution Service (CPS) guidelines on intentional or reckless transmission of infection (England and Wales), the Crown Office and Procurator Fiscal Service (COPFS) guidelines (Scotland), and relevant case law will be detailed.^48^ The extent to which the guidance provided by BHIVA/BASHH accurately reflects current legal approaches to these various issues will also be examined.

### A. Disclosure

The position regarding disclosure must be derived from the limited case law to date and, unfortunately, the precise requirements around disclosure remain somewhat uncertain. While it is clear that there is no absolute legal duty to disclose HIV+ status, it is also evident that failure to disclose can, in some cases, lead to a criminal conviction. The matter is further complicated by the fact that concepts of ‘knowledge’ and ‘consent’ have been linked almost inextricably to the issue of disclosure. With regard to consent, the Court of Appeal in *Dica* recognised that consent by a sexual partner to run the risk of infection could provide a defence to a criminal charge of reckless transmission.^49^ However, in *Konzani*, it was made clear that for consent to operate as a defence, it had to be *informed.*^50^ According to Judge LJ, ‘there is a critical distinction between taking a risk of the various, potentially adverse and possibly problematic consequences of sexual intercourse, and giving an informed consent to the risk of infection with a fatal disease’.^51^ Matthew Weait has taken issue with this distinction, suggesting that it could, in fact, be argued that a person who consents to unprotected sexual intercourse with someone about whose HIV status they are uncertain, consents also to the risk of transmission.^52^ However, by explicitly rejecting this view, the Court of Appeal set a high threshold for informed consent. This threshold largely precludes reliance upon general knowledge of the risks associated with unprotected intercourse or risky sexual behaviour as indicating a willingness to run the risk of infection. Such an approach may undermine the public health message that we are all responsible for our own individual health and it ignores the role played by the non-HIV+ sexual partner in acquiring HIV.^53^

It is possible for consent to be informed, despite a failure by the infected individual to specifically disclose HIV+ status prior to engaging in sexual activity.^54^ In *Dica*, the court held that the ultimate issue was not knowledge but consent, suggesting that in some circumstances the defence of consent might be available notwithstanding a failure to disclose.^55^ This possibility was explicitly recognised in *Konzani*.^56^ However, it was also made clear in that case that concealment of HIV+ status almost inevitably meant that one’s sexual partner had been deceived, and that in such circumstances any consent given would not be properly informed.^57^ The circumstances recognised by the court in *Konzani* where consent could be informed in the absence of disclosure were extremely limited—either an individual with HIV developed a sexual relationship with someone who they met while in hospital receiving treatment for the condition, or the sexual partner was informed of the person’s HIV+ status by a third party.^58^ In these examples, a disclosure is made (either through the context of the hospital setting or through a third party), but not by the infected party.

Moreover, although the courts have recognised the possibility of informed consent in the absence of disclosure by the HIV+ individual, the main thrust of their judgments rest upon an implicit assumption that the source of knowledge for the sexual partner who must consent to the risk of transmission should be the person who may transmit HIV to them.^59^ For example, in *R v Barnes*, Lord Woolf stated that a person would not be guilty under section 20 of the Offences Against the Person Act 1861 for transmitting HIV, if they had made their partner aware of their condition, who (with that knowledge) consented to sexual intercourse because they were prepared to accept the risks involved.^60^ In that case, knowledge and acceptance of risk on the part of the sexual partner was explored entirely through the lens of disclosure by the HIV+ individual.

Furthermore, while an honest belief in consent is recognised as providing a defence to a charge of reckless transmission, it will be extremely difficult to establish such a belief in the absence of disclosure. In *Konzani*, Lord Judge CJ stated that silence, when an individual knows they are infected but does not make their partner aware and engages in unprotected sexual activity, is ‘incongruous with honesty, or with a genuine belief that there is an informed consent’.^61^ Ultimately, it seems that in the absence of direct disclosure by the HIV+ individual it will be relatively easy for the prosecution to prove either that there was no consent to the risk of transmission or no honest belief that such consent existed. It is for this reason that it has been argued by some legal commentators that an ‘effective’ duty to disclose exists in cases of unprotected sexual activity.^62^

The issue of medical advice regarding disclosure has been specifically commented on in a number of cases.^63^ In *Konzani*, a case involving unprotected sexual intercourse with multiple partners, it was relied upon by the prosecution as a way of constructing Konzani’s recklessness and, ultimately, his guilt. The prosecution pointed out that:

… he was specifically advised, wasn’t he, about a number of things: …. the risk that he posed to others. He was specifically told that he must always have safe [*sic*] sex and he was also specifically advised, wasn’t he, and you will remember this, that he should tell people he was going to have sex with. He must tell these people that he was HIV positive.^64^

The Court of Appeal in *Konzani* highlighted the connection between recklessness, consent, and disclosure; making it clear that non-disclosure itself may be viewed as reckless or at least be considered part of the reckless behaviour. It also clarified that the accused in *Dica* had been reckless because he had knowledge of his HIV+ status, knowledge regarding modes of transmission, and had concealed his condition from his partner.^65^ Weait suggests that this is a ‘radical interpretation of recklessness’ as it extends beyond ‘conscious, unjustifiable risk-taking’ to include the ‘additional element of non-disclosure’.^66^

In *Mola*, the medical advice given was used to partly exculpate the accused’s non-disclosure but also to support a finding of guilt for failure to wear a condom.^67^ Mola had received medical advice to the effect that he did not need to disclose his HIV+ status or hepatitis C infection to his sexual partners provided that he used condoms for penetrative intercourse. Lord Hodge found that it was not possible to find that Mola’s non-disclosure was, in and of itself, reckless in the light of that medical advice.^68^ It seems that Lord Hodge may have had some doubt regarding the advice given, but he declared that ‘It is not for me to judge whether the advice you were given was appropriate’.^69^ Mola was found guilty of culpable and reckless conduct because he had failed to wear a condom during sex, despite the clear medical advice to do so. Similarly, during the sentencing of *Devereaux*, Lord Pentland commented that:

You were well aware from the medical advice given to you that you were at risk of infecting any sexual partner if you had unprotected intercourse, but you chose not to inform any of your partners that you had the virus and you chose not to use a condom or take any precautions.^70^

Here, again, there is a reference to non-disclosure, failure to use precautions, and a refusal to follow medical advice provided, and these combine to establish reckless and culpable conduct on the part of the accused.

The BHIVA/BASHH guidance that disclosure is not required in the context of low risk activity such as condom use or low viral load (achieved through adherence to ART medication) has not specifically been dealt with by the English and Welsh, or Scottish courts. As outlined above, the contested cases have dealt with the issue of non-disclosure in the context of high-risk activity (unprotected sexual intercourse) and in these circumstances non-disclosure is highly likely to be deemed reckless. Low viral load or condom use may be of significance in determining recklessness (discussed further below); however, the relationship between the risk of transmission and disclosure requirements in domestic law remains unclear. Interestingly, in Canada, the disclosure obligation has been considered on the basis of the risk of transmission. It was held in *R v Cuerrier*, that disclosure was only required where there was a significant risk of transmission.^71^ This was subsequently clarified in *Mabior*, where the court held that disclosure was required where there was a realistic prospect of transmission.^72^ In its Consultation Paper on Offences Against the Person, the Law Commission of England and Wales identified three possible approaches to disclosure in the context of liability for HIV transmission.^73^ One, echoing the Canadian approach established in *Cuerrier*, was that disclosure would only be required where there was a significant risk of transmission. The other possibilities were that disclosure would be required in all circumstances, or the matter of non-disclosure would be left as a jury question in each case. The Law Commission ultimately decided that a wider review of the issue was needed, and did not come to any firm conclusion on the way to deal with non-disclosure.^74^

### B. Recklessness and condom use

Under English criminal law, a person is reckless if they are aware of a risk and take it, and it is, in the circumstances, unreasonable to take that risk.^75^ The notion of risk here can be understood in two ways. First, it can refer to the likelihood of harm occurring. The criminal law is generally not concerned with negligible risks, although sometimes quite small risks may be punished if there is no social value attached to the conduct which creates the risk.^76^ The second way in which risk can be understood is with regard to seriousness of the risk. In this specific context, it is a consideration of the seriousness of HIV infection for an individual, as opposed to the likelihood that HIV will be transmitted that is important. The more serious the harm someone is exposed to, the harder it can be to justify taking a risk of such harm. The issue of reasonableness or justifiability of risk is one that is generally left unexplored in the criminal law.^77^ When it is considered, the question of justifiability or reasonableness of risk is judged objectively and is ultimately decided by the jury in a contested case.^78^

As discussed in Section II, there is a wealth of medical evidence that shows that proper use of condoms significantly reduces the risk of HIV transmission. When it comes to considering whether a person was reckless in a legal sense, it could be argued either that condom use renders risk-taking justifiable or that it reduces the risk of transmission to such an extent that the risk is no longer legally significant. Alternatively, it could be argued that condom use should give rise to a safer sex defence to someone charged with transmitting HIV. In *Dica*, Judge LJ observed that if protective measures had been taken, this would be a factor to be considered (together with all other relevant evidence) by the jury when determining whether recklessness on the part of the accused was established.^79^ The CPS guidelines for England and Wales provide that:

Evidence that the suspect took appropriate safeguards to prevent the transmission of their infection throughout the entire period of sexual activity, and evidence that those safeguards satisfy medical experts as reasonable in light of the nature of the infection, will mean that it will be highly unlikely that the prosecution will be able to demonstrate that the suspect was reckless.^80^

Similarly, the COPFS guidelines in Scotland provide that the fact that the person infected took appropriate precautions, such as using a condom or other safeguards throughout the sexual activity, will mean that it is unlikely that the appropriate degree of recklessness will be established.^81^

Yet these statements must be interpreted carefully. Neither unequivocally states that a person who uses precautions is not reckless. Instead, they indicate that use of a condom will be important evidence when determining whether someone has been reckless. This may seem like a fine distinction, but it is a distinction with significance as it allows for the possibility of a finding of recklessness notwithstanding the use of precautions. It is also worth noting that neither of the statements suggest that condom use renders the level of risk so low as to be beneath the threshold required for criminal liability; rather, attention remains on the mental element (*mens rea*) required for criminal liability—recklessness.

Under current criminal law, it is entirely possible that liability could be imposed even when condoms have been used. The use of a condom could be treated as demonstrating a person’s awareness of the risk of transmission and thereby help establish the *mens rea* of recklessness, rather than negate it. As David Ormerod and Michael Gunn have recognised, a person using precautions is still a conscious risk taker and may, therefore, be viewed as reckless.^82^ However, in the light of the prosecutorial guidelines regarding condom use and recklessness in England and Wales, and Scotland, it is *probably* unlikely (but note the lack of certainty) that such an approach would be taken.^83^ This would tend to suggest that the BHIVA/BASHH position on condom use preventing a finding of recklessness is broadly in line with the legal position.

Similar to the BHIVA/BASHH guidelines, the CPS guidelines state that when safeguards (such as condom use) cease to be effective, the suspect should disclose their infection status to their sexual partner so that they can choose whether to assume the risk and thereby provide a defence to the suspect.^84^ It therefore appears that disclosure is not required under the CPS guidelines *provided* that effective safeguards are taken, and that if effective safeguards have been used, then a person will not be reckless. In Canadian law, the issue of condom use, risk, and disclosure has been addressed directly by the courts. In the case of *Cuerrier*, the Candian Supreme Court stated that careful use of condoms reduced the risk of harm to such an extent that the risk could no longer be considered significant and, therefore, fell below the threshold for criminal sanction.^85^ Disclosure was thus not required if the HIV+ person ensured proper and consistent condom use. Although it is worth noting that this position was modified in the subsequent case of *Mabior*, where it was held that condom use alone did not in fact preclude a realistic possibility of transmission.^86^

The uncertainty surrounding condom use and the potential for criminal sanction in this jurisdiction must be recognised. Although recklessness would not be an issue in England and Wales if transmission had not in fact occurred, if transmission occurred despite the use of precautions and a case went to trial, the question of whether condom use negated a charge of recklessness in circumstances of non-disclosure, would be decided by the jury (in the absence of a clear legal ruling or legislation on the issue). A jury may well be uncomfortable with this kind of unilateral decision-making or risk-taking, especially if HIV transmission had occurred, and they may well believe that a sexual partner has a right to know, even in the context of safer sex.^87^ It is also likely that arguments would be made regarding the validity of the consent given to sexual activity in such circumstances; that consent could not be said to be truly informed without disclosure. So while it seems that criminal charges would not be pursued if there was evidence of consistent and effective condom use, this is currently dependent on prosecutorial discretion and the matter becomes much less certain should a case go to trial because transmission had taken place. It would, therefore, be preferable to have a definitive statement regarding the use of condoms negating criminal liability or providing a defence to a charge of reckless HIV transmission. This could take the form of clearer prosecution guidelines, although a legal ruling to this effect, or new legislation, might offer more certainty. It would also place the BHIVA/BASHH guidelines on condom use on a more secure footing.

### C. Low viral load

The issue of low viral load and its relationship to recklessness and disclosure has not yet been subject to a legal ruling in England and Wales or Scotland. It is, however, considered in the CPS and COPFS guidelines.^88^ The BHIVA/BASHH guidelines provide that adherence to ART medication leading to low viral load would mean that a HIV+ person would avoid prosecution for reckless transmission.^89^ It is said that ‘in some situations an undetectable viral load can afford protection equivalent to, or greater than that of condoms’.^90^ The CPS prosecution guidelines treat the issue of low viral load with some circumspection. Although recognising that adherence to treatment resulting in an undetectable viral load significantly reduces the risk of transmission, the CPS guidance observes that medical opinion on the reduction of risk is not yet finalised, and that evidence of taking medication in accordance with medical advice may not be as clear cut as evidence of the use of other safeguards, such as condoms.^91^ It therefore suggests that the issue of viral load should be approached with caution.^92^ The reason for the divergence here may simply be that the CPS guidance, first issued in 2011, had already become somewhat dated by the time of the BHIVA/BASHH 2013 recommendation. The Scottish guidelines, issued in July 2014 note that there is a body of medical opinion that there is minimal or negligible risk of transmission when plasma viral load is below 50.^93^ It further states that it will be difficult to establish the requisite recklessness when the person infected is receiving treatment which effectively suppresses viral load and has been given medical advice that there is a low risk of transmission.^94^

The issue of low viral load has been considered by the Supreme Court of Canada in *Mabior*.^95^ In the earlier case of *Cuerrier*, which considered whether disclosure was required if someone was using precautions, the court held that the extent of the duty to disclose was linked to the level of risk of transmission.^96^ Furthermore, where the risk of bodily harm was not significant, as in the case of careful and consistent use of condoms, the duty to disclose did not arise. In *Mabior*, the Court of Appeal held that *either* low viral load *or* condom use would mean that the risk of bodily harm was not significant.^97^ However, the Canadian Supreme Court adopted a different position. It held that low viral load on its own was not enough to prevent criminal liability; it was only when low viral load was combined with condom use that the risk of transmission could be considered negligible and not sufficient to attract criminal sanction.^98^ Non-disclosure was, therefore, only sanctioned in the context of condom use together with low viral load.

It has been suggested that by requiring both condom use and low viral load the Canadian Supreme Court in *Mabior* was essentially equating realistic possibility of transmission with no possibility of transmission.^99^ Of course, at the time of the *Mabior* decision in 2012, the scientific research on level of risk and low viral load was only beginning to emerge.^100^ The Canadian Supreme Court was, however, cognisant of the claims around level of risk and low viral load, and stated that the legal position on low viral load set out in their decision might have to be adapted in future in line with advances in treatment and management of HIV infection.^101^ Attempts have been made to bring the realistic possibility of transmission test more into line with the prevailing scientific findings. For example, the Department of Justice Canada, in its 2017 report, ‘Criminal Justice System’s Response to Non-disclosure of HIV’ stated that:

the criminal law should not apply to persons living with HIV who have engaged in sexual activity without disclosing their status if they have maintained a suppressed viral load (i.e., under 200 copies per ml of blood), because the realistic possibility of transmission test is not met in these circumstances.^102^

This position reflects the scientific assessment of risk as set out in the PARTNERS2 study (discussed in Section II).^103^ In 2018, the Federal Attorney General issued a binding directive to the Public Prosecution Service of Canada (PPSC), that HIV non-disclosure cases where the person living with HIV has maintained a suppressed viral load (under 200 copies per ml of blood), should not be prosecuted.^104^ This Federal Directive only limits prosecutions in three territories (Yukon, Northwest Territories, and Nunavut), it being the responsibility of the provincial Attorney Generals in the other provinces to set prosecutorial guidance.^105^ Given this, the Canadian HIV/AIDS Legal Network has argued that to avoid unjust prosecutions in other Canadian provinces it is essential that similar directives or guidance on low viral load be issued.^106^

Due to the lack of a legal ruling on the issue of low viral load and its relationship to recklessness and disclosure obligations in England and Wales, and Scotland, the exact legal position remains unclear and it cannot be said for certain that the position laid out in the BHIVA/BASHH guidelines is legally accurate. The CPS guidelines on the issue of low viral load take a more cautious approach to low viral load than that in the BHIVA/BASHH guidelines; although, arguably, the CPS guidelines are outdated and should be changed to reflect current scientific understanding of risk.^107^ The high threshold set in the COPFS guidelines regarding low viral load (plasma viral load below 50 as compared with viral load below 200 as seen in much of the research) could also be viewed as taking an overly cautious approach and should be reconsidered.^108^ It is recognised that these concerns could be viewed as largely hypothetical if U really does equal U, as transmission will not occur. Yet for those living with HIV, as well as those treating people with HIV and those who may have to consider potential criminal charges, greater clarity (either by virtue of a legal ruling, legislation or updated prosecutorial guidelines) regarding the relationship between low viral load and the issue of recklessness, and regarding disclosure obligations in the context of low viral load and how this relates, in law, to the issue of consent is needed.

## IV. NON-DISCLOSURE AND THE BOUNDARIES OF MEDICAL ADVICE

The BHIVA/BASHH guidelines arguably stray beyond the normal parameters of medical advice by both presenting as a form of legal advice and by offering what could be viewed as a moral judgement as to when disclosure is required. First, the guidelines are framed specifically in terms of avoiding criminal prosecution for reckless transmission and, therefore, offer what could be seen as a form of legal advice.^109^ This seems to go further than the advice suggested later in the BHIVA/BASHH guidelines under the heading ‘Advice that should be provided by the clinical team to all patients diagnosed with HIV infection’.^110^ In that section, it is said that people with HIV should be advised that there have been successful prosecutions for reckless transmission of HIV.^111^ This latter position seems to allow for general awareness raising regarding criminalisation. However, the offering of medical advice which starts from the premise ‘to avoid prosecution’ seems to go beyond mere awareness raising. While it is recognised that healthcare professionals need to be aware of the law on HIV transmission, and that the subject matter will often have to be addressed with patients, it is important that such conversations remain clear with regards to what has been *decisively* settled in the law on HIV transmission. Uncertainties in the law may be highlighted, but it is important that healthcare professionals do not make assumptions about likely approaches to criminal liability. Moreover, exchanges between healthcare professionals and their patients about potential criminal liability should not be seen as a substitute for legal advice, nor should healthcare professionals offer assurance that particular behaviours will not fall foul of the law when that has not, in fact, been tested in the courts. The BHIVA/BASHH guidelines, framed as they currently are, place healthcare professionals in a potentially precarious position, as the advice on criminal liability rests upon views about risk and liability that are only partly reflected in the prosecutorial guidelines and have not yet been settled in law.^112^

Even if the law was clear that disclosure was not required in instances of low transmission risk, it could still be questioned whether professional body guidelines on medical advice for those living with HIV should sanction non-disclosure in any context. James Chalmers has suggested that:

advising that the risk of transmission is significantly reduced by condom use is clearly medical advice, but advising that this reduction means that disclosure is not required is, if anything, moral advice on appropriate standards of behaviour, not medicine.^113^

The BHIVA/BASHH guidelines stray into what Chalmers would interpret as moral judgement on appropriate behaviour. To simply advise that condom use lowers the risk of transmission is a clear, evidenced based, factual statement that serves both to protect the HIV+ individual from further infection (with other STIs) and the wider public from onward transmission. It is a statement that remains neutral regarding disclosure obligations in the context of sexual activity. The same could be said in relation to ART. Advice that adherence to ART and maintaining a suppressed viral load will prevent onward transmission can be communicated without relating this to the issue of disclosure. The effective sanctioning of non-disclosure when the risk of transmission is low or non-existent, does, on the face of it, seem to stretch the concept of medical advice by going beyond the information that is needed to restore or preserve health or to protect others.^114^ It also has the potential to undermine the position expressed in the BHIVA/BASHH guidelines that people with HIV should disclose their positive status to sexual partners before sexual activity, so as to support informed agreement around risk and safe sex behaviours.^115^

It has been suggested that this apparent blurring of medical advice to the HIV+ individual with guidance regarding appropriate behaviour with others, is, at least in the area of sexual health, impossible to avoid.^116^ For those involved in sexual health work, a duty is not only owed to the patient but also to the wider public (a public health duty aimed at preventing or limiting the transmission of STIs).^117^ This means that sexual health professionals are frequently required to balance the rights and interests of the HIV+ patient with those of the wider community.^118^ As Kilian Dunphy has recognised, sexual health professionals routinely give information ‘on levels of risk of causing degrees of harm’ and identify circumstances in which ‘we (the health professionals) feel that risk not justified’.^119^ This involves ‘a quintessentially moral judgement’, which is an intrinsic part of the health professional’s role in this context.^120^ While this may be true, this does not necessarily answer the question whether healthcare professionals should be indicating that disclosure is not required in cases of low or no risk of transmission. To echo Chalmers, it is one thing to tell a HIV+ person that condom use or low viral load will mean a sexual partner will be protected from transmission and that this is responsible and appropriate behaviour, it is another thing altogether to suggest that there is no need to disclose HIV status to that partner.

It must also be recognised that divergent views exist amongst healthcare professionals regarding disclosure obligations. Research conducted by Catherine Dodds and colleagues, which explored HIV service providers’ perspectives on the criminalisation of transmission, found that some of the healthcare professionals consulted believed there was a moral obligation to disclose, regardless of the risk that a particular sexual encounter might carry.^121^ Furthermore, the law was often used by HIV service providers ‘as an externalised tool to help dispel their moral unease’ about non-disclosure.^122^ These providers promoted HIV status disclosure as the only means of avoiding criminal liability.^123^ Others expressed views that were more reflective of the BHIVA/BASHH position and rejected a universal disclosure obligation, believing that where someone actively sought to reduce the chance of transmission (either through condom use or the maintenance of a low viral load) they should not be criminally charged.^124^ However, it is unclear if these views regarding the appropriate boundaries of criminal liability translated into the provision of advice that disclosure would not be required in low risk activity. In broader terms, it is worth noting that, guidelines notwithstanding, divergent advice on disclosure was being provided by those who took part in the study.^125^

The fact that the BHIVA/BASHH position on non-disclosure prompts questions about the moral basis of disclosure obligations, the rights of the sexual partner to determine for themselves the level of risk they are willing to accept, and whether the approach to disclosure should be based solely on the scientific assessment of risk, raises questions as to whether the guidance strays beyond straightforward medical advice about how to preserve one’s health and prevent onward transmission.^126^ If the guidance does, in fact, extend beyond the parameters of normal medical advice, it is important to consider whether it is acceptable for such advice to be given by healthcare professionals. To some extent, it may seem incongruous for those who have a responsibility to protect others to sanction non-disclosure, and it could be questioned whether by doing so healthcare professionals are failing in their duties to the wider community. Yet, the argument can be made that the duty is to protect others from the *risk* of transmission, and given that no risk or a very low risk of transmission exists in cases of low viral load or condom use, this public health duty is not engaged. It may also be possible to justify the BHIVA/BASHH view on non-disclosure by focusing on the clinical utility of disclosure. On this premise, disclosure is not required if HIV cannot be transmitted because the purpose of disclosing a condition is to enable sexual partners to make effective decisions about prevention and safe sex. If a condition cannot be transmitted, then that purpose no longer exists. It is suggested that both justifications, risk and clinical utility, are at their strongest in the context of low viral load where no risk of transmission is said to exist. They may be less convincing in the context of condom use, where a risk, albeit a low one, remains and it could be thought that the sexual partner should be able to determine what level of risk they are willing to take.^127^

While questioning the appropriateness of medical advice effectively sanctioning non-disclosure in the context of low transmission risk, it is recognised that when determining what the law regarding HIV transmission should be, a case could be made that non-disclosure in the context of condom use or low viral load should not be subject to criminal sanction.^128^ It is not possible to fully set out the reasons in support of this approach to criminalisation here due to limitations of space; however, a brief account of the essential argument can be given.^129^ Both HIV+ individuals and their sexual partners can invoke autonomy, bodily integrity and privacy arguments in relation to disclosure obligations. For the HIV+ individual, it could be argued that to require disclosure where the risk of transmission is low or non-existent would be a disproportionate interference with their right to autonomy, privacy and sexual freedom.^130^ Although these rights are not absolute and must be balanced against the rights and interests of the sexual partner, concerns around confidentiality and stigma arguably help tip the balance in favour of protecting the HIV+ individual’s autonomy and privacy when the risks of transmission are low.^131^

Sexual partners can also base an argument for disclosure on autonomy and bodily integrity, it being claimed that disclosure is necessary to enable an informed, autonomous choice about the risks they are willing to take.^132^ This view may be challenged on a number of grounds, two will be highlighted here. First, a failure to disclose does not necessarily preclude an autonomous choice.^133^ A sexual partner can have knowledge regarding HIV transmission risk even in the absence of direct disclosure by the infected individual and make their choice about whether to engage in sexual activity accordingly.^134^ Some would contend that knowledge of infected status places greater responsibility on the HIV+ individual.^135^ Yet, it is arguable that this responsibility to sexual partners can be met *either* through disclosure *or* by ensuring sexual activity only takes place in the context of low or negligible risk of transmission.^136^ Secondly, and more to the point for our present purposes, in the context of low viral load and condom use, the level of risk may be deemed so low as to not engage a disclosure requirement for the purposes of considering recklessness or consent to risk; the risk could be judged to be legally insignificant.^137^ To allow condom use or low viral load to negate a charge of reckless transmission or exposure, even without disclosure, arguably strikes the appropriate balance between the privacy and autonomy interests of the HIV+ individual and the autonomy and bodily integrity interests of their sexual partner.

Ultimately, it is not suggested that the BHIVA/BASHH overarching position on non-disclosure in the case of low risk is indefensible. Indeed, as just highlighted, the criminal law could justifiably provide that disclosure is not required when condoms are used or the infected person has a low viral load. What is questioned is whether it is appropriate for medical advice to offer an assurance that this is a way to avoid criminal prosecution when that has not yet been fully established in law. The effective sanctioning of non-disclosure to sexual partners in the guidelines (even when limited to cases of low or negligible risk) is also challenged. Green-lighting of non-disclosure by healthcare professionals seems to offer a view regarding disclosure obligations that strays into a moral assessment of appropriate behaviour and goes beyond our normal understanding of medical advice, which is focused on providing information needed to maintain and preserve the health of the HIV+ individual and protect others from onward transmission.

## V. CONCLUSION

The BHIVA/BASHH guidelines provide that to avoid criminal prosecution the HIV+ individual should use condoms, maintain a suppressed viral load or otherwise disclose their condition.^138^ It has been suggested that these guidelines stray beyond the boundaries of ‘normal’ medical advice (advice which is limited to maintaining, restoring or preserving the health of the HIV+ patient and preventing onward transmission of disease) in two ways. First, the guidelines are explicitly framed as advice on how to avoid prosecution for reckless transmission of HIV.^139^ In this respect, the guidelines appear to offer an assurance regarding potential criminal liability which strays beyond medical professional opinion into the realms of legal advice. Perhaps even more concerning is the fact that the guidelines set out a position on non-disclosure and criminal liability that has not yet been tested in the courts. The current legal position has, unfortunately, developed in an ad hoc fashion, with rules and guiding principles emerging through case law and prosecutorial guidelines, which themselves need updating to take account of current scientific understanding of risk and transmission. Given the potential for criminal sanction, it is submitted that clarity in the law on disclosure requirements and the legal position regarding condom use and low viral load is essential. However, within the confines of the healthcare professional and patient relationship, conversations about potential criminal liability should stay firmly within the confines of awareness raising and be clear regarding what has been decisively settled in the law to date.

Secondly, while legal clarity on disclosure is needed, it remains questionable whether non-disclosure in the case of low transmission risk should be effectively sanctioned within the context of medical advice. This position on non-disclosure contained in the BHIVA/BASHH guidelines has its roots in the scientific understanding of transmission risk, and may also be based on an assessment of the clinical utility of disclosure (if there is no risk of transmission there is no reason or need to disclose). However, by straying beyond informing patients about risk to themselves and others it seems to go beyond medical advice as normally understood, and advance what could be considered a moral view as to appropriate behaviour and disclosure obligations.^140^ Furthermore, it is difficult to reconcile this position on non-disclosure with the statement also made within the professional body guidelines that disclosure is important to enable sexual partners to make informed choices.^141^ It may be that the BHIVA/BASHH guidelines attempt to strike a balance between the various interests at play here (the HIV+ patient and their sexual partner). However, they do so without engaging directly with the wider moral issues around non-disclosure or the rights of the sexual partner. It remains a point for debate as to whether the risk based approach and/or clinical utility view of disclosure should determine the overarching position on disclosure in professional body guidelines, and whether and/or how this is encompassed within medical advice provided to those living with HIV. The importance of disclosure to enable sexual partners make to decisions around safer sex and risk needs further reconciliation with the view that disclosure is not required in the context of low risk.

## Footnotes

1 MD Stein and others, ‘Sexual Ethics: Disclosure of HIV-Positive Status to Partners’ (1998) 158(3) Archives of Internal Medicine 253; V Derlega, B Winstead and L Folk-Barron, ‘Reasons For and Against Disclosing HIV-Seropositive Test Results to an Intimate Partner: A Functional Perspective’ in S Petronio (ed), *Balancing the Secrets of Private Disclosure* (Psychology Press 2014) 53.

2 C Dodds and others, ‘Keeping Confidence: HIV and the Criminal Law from HIV Service Providers’ Perspectives’ (2015) 25(4) Critical Public Health 410; C Evans, ‘The Impact of Criminalising Disease Transmission on the Healthcare Professional-Patient Relationship’ in C Stanton and H Quirk (eds), *Criminalising Contagion* (Cambridge University Press 2016) 78.

3 Evans (n 2) 80.

4 A useful situation to consider here is the duty healthcare professionals have to *encourage* those diagnosed with epilepsy to contact the Driving and Vehicle Licensing Authority (DVLA). Whilst it is the patient’s duty to inform the DVLA of any health condition that may render them unfit to drive, a doctor may breach patient confidentiality and inform the DVLA if the patient does not disclose their condition and continues to drive. The doctor must believe that the patient’s continued driving exposes others to a risk of death or serious injury: General Medical Council (GMC), ‘Confidentiality: Patients’ fitness to drive and reporting concerns to the DVLA or DVA’ <https://www.gmc-uk.org/ethical-guidance/ethical-guidance-for-doctors/confidentiality–-patients-fitness-to-drive-and-reporting-concerns-to-the-dvla-or-dva> accessed 7 March 2021.

5 I Brassington, ‘On the Relationship Between Medical Ethics and the Law’ (2018) 26(2) Medical Law Review 225, 225.

6 J Flaherty, ‘Clarifying the Duty to Warn in HIV Transference Cases’ (2008) 54 Criminal Law Quarterly 60; D Ainslie, ‘AIDS and Sex: Is Warning a Moral Obligation?’ (2002) 10(1) Health Care Analysis 49; MA Field and KM Sullivan, ‘AIDS and the Criminal Law’ (1987) 15 Law, Medicine and Healthcare 46.

7 See GMC, *Good Medical Practice* (GMC 2013, updated in April 2019).

8 See GMC, *Confidentiality: Disclosing Information About Serious Communicable Diseases* (GMC, 2017).

9 M Phillips and M Poulton, ‘HIV Transmission, the Law and the Work of the Clinical Team’ (2013) 25(1) International Journal of STD & AIDS 8, 13.

10 GMC (n 8) 3.

11 ibid 3.

12 Philips and Poulton (n 9) 18.

13 ibid.

14 Philips and Poulton (n 9) 19.

15 ibid.

16 Dodds and others(n 2).

17 Evans (n 2) 78.

18 Philips and Poulton (n 9) 18.

19 Dodds and others (n 2); R Lowbury and GR Kinghorn, ‘Criminal Prosecution for HIV Transmission: A Threat to Public Health’ (2006) 333(7570) BMJ 666; JM Kilty and M Orsini, ‘We Write as Little as We Have to: Charting Practices and Documenting Disclosure in Response to HIV Criminalization in Canada’ (2019) 31(8) AIDS Care 1035.

20 R Lowbury and GR Kinghorn, ‘Criminal Prosecution for HIV Transmission: A Threat to Public Health’ (2006) 333(7570) BMJ 666.

21 Kilty and Orsini (n 19); Dodds and others (n 2).

22 Philips and Poulton (n 9).

23 Dodds and others (n 2). Dodds and colleagues noted that at the time their research was conducted, no audit on the extent to which these guidelines had been disseminated had been conducted.

24 Philips and Poulton (n 9) 11.

25 ibid. Whether this advice remains within the confines of appropriate medical advice will be considered in more detail in Section IV.

26 Philips and Poulton (n 9) 12.

27 SC Weller and KR Davis, ‘Condom Effectiveness in Reducing Heterosexual HIV Transmission’ (2002) Cochrane Database System Review CD003255. This was also expressed as 1.14 transmission per 100-person years. See J LeMessurier and others, ‘Risk of Sexual Transmission of Human Immunodeficiency Virus with Antiretroviral Therapy, Suppressed Viral Load and Condom Use: A Systematic Review’ (2018) 190(46) Canadian Medical Association Journal 1350.

28 I Grant, ‘The Prosecution of Non-disclosure of HIV in Canada: Time to Rethink *Cuerrier’* (2011) 5 McGill Journal of Law and Health 7, 13.

29 E Vittinghoff and others, ‘Per-contact Risk of Human Immunodeficiency Virus Transmission between Male and Sexual Partners’ (1999) 150(3) American Journal of Epidemiology 306.

30 WD Johnson, A O’Leary and SA Flores, ‘Per-partner Condom Effectiveness against HIV for Men Who Have Sex with Men’ (2018) 32(11) AIDS 1499.

31 The importance of clarity regarding the interpretation of data on risk and condom use has been emphasised by, for example, CL Galletly and SD Pinkerton, ‘Toward Rational Criminal HIV Exposure Laws’ (2004) 32 Journal of Law, Medicine & Ethics 327; I Grant, ‘Rethinking Risk: The Relevance of Condoms and Viral Load in HIV Non-disclosure Prosecutions’ (2009) 54 McGill Law Journal 389.

32 See P Patel and others, ‘Estimating Per-Act HIV Transmission Risk: A Systematic Review’ (2014) 28(10) AIDS 1509.

33 SD Pinkerton and PR Abramson, ‘Effectiveness of Condom in Preventing HIV Transmission’ (1997) 44(9) Social Science & Medicine 1303.

34 See, for example, TC Quinn and others, ‘Viral Load and Heterosexual Transmission of Human Immunodeficiency Virus Type 1’ (2000) 342 NEJM 921; J Castilla and others, ‘Effectiveness of Highly Active Antiretroviral Therapy in Reducing Heterosexual Transmission of HIV’ (2005) 40 Journal of Acquired Immune Deficiency Syndrome 96; S Attia and others, ‘Sexual Transmission of HIV According to Viral Load and Antiretroviral Therapy: Systematic Review and Meta-analysis’ (2009) 23 AIDS 1397; D Donnell and others, ‘Heterosexual HIV-1 Transmission After Initiation of Antiretroviral Therapy: A Prospective Cohort Analysis’ (2010) 375 The Lancet 2092; AJ Rodger and others, ‘PARTNER Study – Sexual Activity Without Condoms and Risk of HIV Transmission in Serodifferent Couples When the HIV Positive Partner Is Using Suppressive Antiretroviral Therapy’ (2016) 316(2) JAMA 171; AJ Rodger and others, ‘Risk of HIV Transmission Through Condomless Sex in Serodifferent Gay Couples with the HIV-Positive Partner Taking Suppressive Antiretroviral Therapy (PARTNER): Final Results of a Multicentre, Prospective, Observational Study’ (2019) 393 The Lancet 2428.

35 Rodger and others, ‘PARTNER Study – Sexual Activity Without Condoms’ ibid.

36 ibid 179.

37 Rodger and others, ‘Risk of HIV Transmission through Condomless Sex’ (n 34).

38 Prevention Access Campaign, ’Undetectable = Untransmittable’ <https://www.preventionaccess.org/undetectable> accessed 7 August 2020. For the Consensus Statement see Prevention Access Campaign, ‘Risk of Sexual Transmission of HIV From a Person Living with HIV who has an Undetectable Viral Load’ July 21 2016 <https://www.preventionaccess.org/consensus> accessed 7 August 2020.

39 ‘U=U Taking Off in 2017’ (2017) 4(11) The Lancet e475.

40 BHIVA, ‘BHIVA Encourages Universal Promotion of Universal = Untransmittable (U=U)’ 30 November 2018. <https://www.bhiva.org/BHIVA-encourages-universal-promotion-of-U-U> accessed 7 August 2020.

41 Intentional transmission would be prosecuted under section 18 Offences against the Person Act (OAPA)1861 and reckless transmission under section 20 OAPA 1861. Knowing can include wilful blindness, see JR Spencer, ‘Re-trial for Reckless Infection’ (2004) New Law Journal 762; S Ryan, ‘Reckless Transmission of HIV: Knowledge and Culpability’ [2006] Criminal Law Review 981.

42 Under the Criminal Attempts Act 1981. For attempted transmission, actual transmission need not have occurred but an intention to infect another must be shown.

43 See Crown Office and Procurator Fiscal Service (COPFS), *Intentional or Reckless Sexual Transmission of, or Exposure to, Infection*, 2. <https://www.copfs.gov.uk/images/Documents/Prosecution_Policy_Guidance/Guidelines_and_Policy/Prosecution%20policy%20on%20the%20sexual%20transmission%20of%20infection%20-%20July%2014.pdf> accessed 16 August 2020.

44 Recklessness in the context of the common law offence of culpable and reckless conduct has been defined in the case of *Paton v HM Advocate* (1936) JC 19 as conduct that is ‘gross or wicked, or criminal negligence, something amounting, or at any rate analogous, to a criminal indifference to consequences’.

45 COPFS (n 43) 5 explains that the offence of culpable and reckless conduct covers situations where the conduct does not result in any actual injury but there is danger of injury to others and the accused exhibits criminal recklessness. It is made clear that exposure would only be prosecuted in exceptional circumstances.

46 The first Scottish prosecution was in 2001 when Stephen Kelly was prosecuted for culpable and reckless conduct for transmitting HIV to his partner. See *HMA v Kelly* (Unreported) 23 February 2001. The first prosecution in England and Wales in 2004 was of Mohammed Dica, who was convicted of recklessly transmitting HIV to two of his sexual partners. See *R v Dica* [2004] 2 Cr App R 28.

47 For example, *Daryll Rowe* (Unreported) Lewes Crown Court 15 November 2017 (intentional transmission of HIV); *R v Dica* [2004] 2 Cr App R 28; *R v Konzani* [2005] 2 Cr App R 14 (reckless transmission of HIV); *R v Golding* [2014] EWCA Crim 889 (reckless transmission genital herpes); *HMA v Mola* (Unreported) 7 February 2007 (culpable and reckless transmission of HIV and hepatitis C); *Ercan Yasar* (Unreported) Gloucester Crown Court 14 November 2008 (hepatitis B and chlamydia); *R v Marangwanda* [2009] EWCA Crim 60 (reckless transmission of gonorrhoea); *HMA v Deveraux* (Unreported) 25 Feb 2010 and *HMA v Daryll Rowe* (Unreported) 4 May 2018 (HIV transmission and exposure).

48 Crown Prosecution Service (CPS), *Intentional or Reckless Sexual Transmission of Infection: Legal Guidance*. <https://www.cps.gov.uk/legal-guidance/intentional-or-reckless-sexual-transmission-infection> accessed 16 August 2020; COPFS (n 48).

49 [2004] 2 Cr App R 28.

50 [2005] 2 Cr App R 14.

51 [2005] 2 Cr App R 14, [41]. Similar requirements for informed consent are found in the CPS and COPFS prosecutorial guidelines (n 48). The COPFS guidelines state that it is highly unlikely that the requisite standard of criminal recklessness would be achieved in circumstances where the victim gave their informed consent and free agreement to sexual activity in the knowledge of the risk of transmission of infection. COPFS (n 48) 8.

52 M Weait, ‘Knowledge, Autonomy and Consent: *R v Konzani*’ [2005] Criminal Law Review 763, 765.

53 CL Galletly and SD Pinkerton, ‘Conflicting Messages: How Criminal HIV Disclosure Laws Undermine Public Health Efforts to Control the Spread of HIV’ (2006) 10(5) AIDS Behaviour 451.

54 This is recognised in the CPS guidelines, which state that ‘informed consent does not necessarily mean that the suspect has disclosed his condition’: CPS (n 48).

55 [2004] 2 Cr App R 28, [59].

56 [2005] 2 Cr App R 14, [44].

57 ibid, [42].

58 ibid, [44].

59 V Munro, ‘On Responsible Relationships and Irresponsible Sex – Criminalising the Reckless Transmission of HIV *R v Dica* and *R v Konzani*’ (2007) 19 Child and Family Law Quarterly 112.

60 [2005] 1 Cr App R 30.

61 [2005] 2 Cr App R 14, [42].

62 Weait (n 52) 767.

63 It is also considered in the prosecutorial guidelines. See, for example, COPFS (n 43) 6: ‘In all cases, there must be careful assessment of the exact nature of the medical advice given and whether the advice regarding treatment, medication and precautions to be used, was followed’.

64 As recounted in M Weait, *Intimacy and Responsibility* (Routledge-Cavendish 2007) 47.

65 [2005] 2 Cr App R 14, [41].

66 Weait (n 52) 766.

67 *HMA v Mola* (Unreported) 5 April 2007.

68 ibid.

69 ibid. A copy of the sentencing statement can be accessed here—Microsoft Word—*HMA v Giovanni Mola*—Lord Hodge April 07.doc (bbc.co.uk) accessed 18 March 2021.

70 *HMA v Devereaux* 2010, as reported by BBC News, ‘HIV Man Jailed for Reckless Sex’ 25 February 2010 <http://news.bbc.co.uk/1/hi/scotland/north_east/8519593.stm> accessed 16 August 2020.

71 [1998] 2 SCR 371 (Supreme Court Canada).

72 [2012] SCC 47; [2012] 2 SCR 584, [91].

73 Law Commission, *Scoping Consultation Paper on Reform of Offences Against the Person* No 217 (2014).

74 Law Commission, *Reform of Offences against the Person*, No 361 (2015) para 6.144. The Law Commission did not recommend the creation of a specific offence of failing to disclose—para 6.145.

75 *R v G & Another* [2003] UKHL 50.

76 In assessing whether taking a given risk is justified, a court will consider the magnitude of the risk, the gravity of the potential harm, and the social value of the activity carrying the risk: R Elliott, *Criminal Law & HIV/AIDS: Final Report* (Canadian HIV/AIDS Legal Network and Canadian AIDS Society 1996).

77 For consideration of the justifiability of risk-taking in the context of HIV transmission, see S Ryan, ‘Risk-Taking, Recklessness and HIV Transmission: Accommodating the Reality of Sexual Transmission of HIV Within a Justifiable Approach to Criminal liability’ (2007) 28 Liverpool Law Review 215.

78 G Williams, *Textbook of Criminal Law* (2nd edn, Stevens & Sons 1983) 98.

79 [2004] 2 Cr App R 28, [11].

80 CPS (n 48).

81 COPFS (n 43) 5.

82 DC Ormerod and ML Gunn, ‘Criminal liability for the transmission of HIV’ [1996] 1 Web Journal of Current Legal Issues.

83 CPS (n 48); COPFS (n 43) 5.

84 CPS (n 48); Phillips and Poulton (n 9) 11.

85 [1998] 2 SCR 371, [129].

86 [2012] 2 SCR 584, [94]. To preclude a realistic possibility of transmission, the HIV+ individual had to have a low viral load and use a condom.

87 A similar view can be found in some of the wider commentary; for example, MA Field and KM Sullivan, ‘AIDS and the Criminal Law’ (1987) 15 Law, Medicine and Healthcare 46; DH Ciccarone and others, ‘Sex without Disclosure of Positive HIV Serostatus in a US Probability Sample of Persons Receiving Medical Care for HIV Infection’ (2003) 93 American Journal of Public Health 949.

88 CPS (n 48); COPFS (n 43).

89 Phillips and Poulton (n 9) 11.

90 ibid.

91 CPS (n 48).

92 ibid.

93 COPFS (n 43) 13.

94 ibid.

95 [2012] 2 SCR 584.

96 [1998] 2 SCR 371, [129].

97 [2010] MBCA 93, [87].

98 [2012] 2 SCR 584, [94].

99 See I Grant, ‘The Over-Criminalisation of HIV Transmission’ (2013) 63 University of Toronto Law Journal 475, 482; M Shaffer, ‘Sex, Lies and HIV: Mabior and the Concept of Sexual Fraud’ (2031) 63 University of Toronto Law Journal 466; A Symington, ‘Injustice Amplified by HIV Non-disclosure Ruling’ (2013) 63 University of Toronto Law Journal 485.

100 As discussed in Section II.

101 [2012] 2 SCR 584, [104].

102 Department of Justice Canada, ‘Criminal Justice System’s Response to Non-disclosure of HIV’ (2017) 30 Criminal Justice System's Response to Non-Disclosure of HIV. https://www.justice.gc.ca/eng/rp-pr/other-autre/hivnd-vihnd/hivnd-vihnd.pdf (accessed 20 March 2021).

103 See Rodger and others, ‘Risk of HIV Transmission through Condomless Sex’ (n 34).

104 Attorney General of Canada, ‘Directive to Director of the Public Prosecution Service’, Canada Gazette, Pt I, Vol 152, 8 December 2018, available at Canada Gazette, Pt 1, Vol 152, Number 49: GOVERNMENT NOTICES (accessed 20 March 2021).

105 Canadian HIV/AIDS Legal Network, ‘The Criminalization of HIV Non-disclosure in Canada: Current Status and the Need for Change’ (2019), available at HIV-criminalization-Info-Sheet-1.pdf (hivlegalnetwork.ca) (accessed 20 March 2021).

106 ibid 4.

107 CPS (n 48).

108 COPFS (n 43) 13.

109 Phillips and Poulton (n 9) 11.

110 ibid 17/18.

111 ibid 18.

112 Phillips and Poulton (n 9)

113 J Chalmers, ‘Getting Mixed Up in Crime: Doctors, Disease Transmission, Confidentiality and the Criminal Process’ in D Griffiths and A Saunders (eds), *Bioethics, Medicine and the Criminal Law* Vol 2 (Cambridge University Press 2013), 77.

114 The BHIVA/BASHH guidelines note that advice given to HIV+ patients must include the routes of HIV transmission and how to prevent transmission, with information about safer sexual practices, the use of condoms and suppression of viral load. Phillips and Poulton (n 9) 11. This seems to be closer to Chalmers understanding of medical advice – J Chalmers (n 113).

115 Phillips and Poulton (n 9) 11.

116 See KP Dunphy, ‘Sexual Rights and the Public Health: Why Moral Judgements Matter’ (2017) 93(2) Sexually Transmitted Infections 83. See also C Cook, ‘The Sexual Health Consultation as a Moral Occasion’ (2014) 21(1) Nursing Inquiry 11.

117 GMC (n 8) 3 notes that a healthcare professional may disclose information to a close contact of someone with a serious communicable disease if they believe that person to be at risk of serious harm and the patient has not informed them and cannot be persuaded to do so. See also Phillips and Poulton (n 9) 13–14.

118 Much of this balancing has, in the past, focused on the issue of confidentiality. See, generally, J Chalmers*, Legal Responses to HIV and AIDS* (Hart Publishing 2008).

119 KP Dunphy, ‘Sexual Rights and the Public Health: Why Moral Judgements Matter’ (2017) 93(2) Sexually Transmitted Infections 83.

120 ibid.

121 C Dodds and others (n 2) 415.

122 ibid 421.

123 ibid 414.

124 ibid 415.

125 Dodds and others (n 2).

126 Chalmers (n 113).

127 See D Warburton, ‘A Critical Review of English Law in Respect of Criminalising Blameworthy Behaviour by HIV+ Individuals’ (2004) 68 Journal of Criminal Law 55, 65 states that ‘even intercourse with the use of a prophylactic barrier in these circumstances is arguably something that most people would shy away from’. D Ciccarone and others, ‘Sex without Disclosure of Positive HIV Serostatus in a US Probability Sample of Persons Receiving Medical Care for HIV Infection’ (2003) 93 American Journal of Public Health 949, argue that the disclosure is essential so that the sexual partner can make an informed choice about what level of risk is acceptable to them.

128 For discussions about criminalisation that reflect on the issue of low transmission risk, see M Cornett, ‘Criminalisation of the Intended Transmission or Knowing Non-disclosure of HIV in Canada’ (2011) 5(1) McGill Journal of Law and Health 61; I Grant, ‘The Boundaries of the Criminal Law: The Criminalisation of the Non-disclosure of HIV’ (2008) 31 Dalhousie Law Journal 123l D Hughes, ‘Condom Use, Viral Load and the Type of Sexual Activity as Defences to the Sexual Transmission of HIV’ [2013] Journal of Criminal Law 136.

129 See S Ryan, ‘Active Deception versus Non-Disclosure: HIV Transmission, Non-Fatal Offences and Criminal Liability’ [2019] Criminal Law Review 1; S Ryan, ‘Disclosure and HIV Transmission’ [2015] Journal of Criminal Law 395; S Ryan, ‘Risk-Taking, Recklessness and HIV Transmission: Accommodating the Reality of Sexual Transmission of HIV within a Justifiable Approach to Criminal Liability’ (2007) (28) Liverpool Law Review 215.

130 S Ryan, ‘Disclosure and HIV Transmission’ [2015] Journal of Criminal Law 395.

131 ibid.

132 L Cherkassky, ‘Being Informed: The Complexities of Knowledge, Deception and Consent When Transmitting HIV’ (2010) Journal of Criminal Law 242; L Gostin and J Hodge, ‘Piercing the Veil of Secrecy in HIV/AIDS and Other Sexually Transmitted Diseases: Theories of Privacy and Disclosure in Partner Notification’ (1998) 5(9) Duke Journal of Gender Law and Policy 9.

133 R Bennett, H Draper and L Frith, ‘Ignorance Is Bliss? HIV and Moral Duties and Legal Duties to Forewarn’ (2002) 26 Journal of Medical Ethics 9; M Weait, ‘Criminal Law and the Sexual Transmission of HIV: *R v Dica*’ (2005) 68(1) Modern Law Review 121.

134 Weait, ibid.

135 See G Mawhinney, ‘To Be Ill or to Kill: The Criminality of Contagion’ (2003) Journal of Criminal Law 202, 203. Mawhinney argues that it is solely the responsibility of those who are ill to disclose their HIV+ status.

136 For example, the CPS guidelines provide that where one partner knows there is a risk of transmitting an infection and the other does not know, it is reasonable to place the major share of responsibility for avoiding the risk upon the partner who knows. It seems possible that this responsibility can be met through the use of precautions and not only through disclosure. CPS (n 48).

137 Law Commission, *Reform of Offences against the Person: A Scoping Consultation Paper No 217* (2014), para 6.92: ‘consent to intercourse implies consent to the normal level of risk, and in the cases with which we are concerned [use of condom and undetectable viral load] the level of risk is no more than normal, and may be less’.

138 Philips and Poulton (n 9) 11.

139 ibid.

140 Chalmers (n 113).

141 Philips and Poulton (n 9) 11.

